# Identification of U6 Promoter and Establishment of Gene-Editing System in *Larix kaempferi* (Lamb.) Carr

**DOI:** 10.3390/plants14010045

**Published:** 2024-12-26

**Authors:** Jun-Xia Xing, Ao-Jie Luo, Xin-Hao Wang, Qi Ding, Ling Yang, Wan-Feng Li

**Affiliations:** 1 State Key Laboratory of Tree Genetics and Breeding, College of Forestry, Northeast Forestry University, Harbin 150040, China; junxiaxing@nefu.edu.cn; 2 State Key Laboratory of Tree Genetics and Breeding, Key Laboratory of Tree Breeding and Cultivation of the National Forestry and Grassland Administration, Research Institute of Forestry, Chinese Academy of Forestry, Beijing 100091, China; luo.aj@caf.ac.cn (A.-J.L.); wxh@caf.ac.cn (X.-H.W.); 3 Life Science and Technology Center, China National Seed Group Co., Ltd., Wuhan 430073, China; qi.ding@syngentagroup.cn; 4 College of Forestry, Beijing Forestry University, Beijing 100083, China

**Keywords:** CRISPR/Cas9, *Larix*, *SCL6*, sgRNA

## Abstract

This study aimed to establish a CRISPR/Cas9 gene-editing system for *Larix kaempferi* (Lamb.) Carr. (Japanese larch). We screened *L. kaempferi* U6 promoters and used them to drive sgRNA expression in the CRISPR/Cas9 gene-editing system. The *L. kaempferi* embryogenic callus was used as the receptor material for genetic transformation, and the frequency and types of gene editing were then analyzed. The results showed various mutations in the transgenic materials, including base substitutions and deletions, and the editing frequency ranged from 5% to 14.29%. In summary, we established a CRISPR/Cas9 gene-editing system for *L. kaempferi*. Our results demonstrate that the CRISPR/Cas9 system can efficiently edit genes in *L. kaempferi*, with significantly higher editing frequencies observed when sgRNA expression is driven by endogenous *LaU6* promoters compared to the exogenous promoter ProAtU6-26. This work provides technical support for the study of *L. kaempferi* gene functions and genetic improvement.

## 1. Introduction

*Larix kaempferi* (Lamb.) Carr. (Japanese larch) is a prevalent tree species in the boreal forests of China with significant economic and ecological value, and the current system for the regeneration and genetic transformation of *L. kaempferi* [1] can be used to improve many traits with economic and ecological importance, such as resistance to biological and abiotic stress [2,3,4], and sterility [5]. However, the lack of efficient genetic manipulation tools has greatly impeded research on the production of new varieties of *L. kaempferi* with desirable characteristics.

Genome editing is used to efficiently modify biological traits or investigate gene function. Since its development in 1987, the CRISPR/Cas system has become a powerful tool for gene editing [6]. Notably, the CRISPR/Cas9 system is characterized by its simplicity, stability, and versatility [7,8,9] and is the most widely used gene-editing tool in plants. This system consists of two basic components, Cas9 nuclease and single-guide RNA (sgRNA). In order for the system to function, it must carry out three processes: expression, interference, and adaptation [10,11]. During the expression stage, sgRNA binds to the Cas9 protein to form a complex and is directed to the target sequence after the protospacer adjacent motif (PAM) for interference. The target sequence is double-stranded and cleaved by the nuclease domains (HNH and RuvC) of the Cas9 protein [10,11,12]. Then, the broken double-strands are left behind as the Cas9 complex detaches, which is attached to the cut end of the target DNA via homologous directed repair at the end of the interference. With continuous improvements and upgrades, this system has become progressively more powerful and has been applied in an increasing number of experimental research studies, practical production, and breeding.

At present, the CRISPR/Cas9 system has been successfully applied in many species, including *Arabidopsis* [13,14], tobacco [15], poplar [16], apple [17], wheat [18], and rice [19]. Excitingly, CRISPR/Cas9-mediated targeted mutagenesis has been realized in other conifers [20,21,22]. Yoshihiko Nanasato et al. successfully knocked out the *Cryptomeria japonica* D. Don magnesium *chelatase subunit I* gene, which is required for chlorophyll biosynthesis in *C. japonica*, causing the plant material to exhibit a white or light green phenotype [20]. Cui Ying et al. used PgCas9/PaU6 to edit the endogenous *1-deoxy-D-xylulose-5-phosphate synthase 1* gene in *Picea glauca* and successfully obtained albino plants [21]. Charleson Poovaiah et al. successfully edited the *glucuronic acid substitution of the xylan 1* gene in *Pinus radiata* (D. Don) [22]. The successful application of CRISPR/Cas9 technology in conifers provides a solid foundation for the development of gene-editing technology in *L. kaempferi*. However, gene-editing technology has not yet been used on a large scale in larch. Recently, Ma Miaomiao et al. designed two target sites in the exon of the *phytoene desaturase* gene and successfully obtained gene-edited somatic embryo seedlings using the particle bombardment method, with a gene editing efficiency of 1.423–2.136% [23]. Compared with the particle bombardment method, *Agrobacterium*-mediated genetic transformation has higher transformation efficiency and more stable expression [24]. Therefore, the improvement of gene editing technology with *Agrobacterium*-mediated genetic transformation is of great significance for larch to breed high-quality lines on a large scale.

In our previous studies, Zhang Shougong et al. analyzed miRNA expression profiles in embryonic and non-embryonic callus of *L. kaempferi* and found that microRNA171 (miR171) was up-regulated [25], and Li Wan-Feng et al. found that *L. kaempferi SCARECROW-LIKE6* (*LaSCL6*) was the target gene of miR171 [26]. The expression of *LaSCL6* can be detected in callus and different organs of seedlings, and its levels change during somatic embryogenesis [27], indicating that the miR171-*LaSCL6* module regulates somatic embryogenesis. So, it is important to study the roles of the miR171-*LaSCL6* module in the somatic embryogenesis of *L. kaempferi*. Therefore, in this study, *LaSCL6* was used as the target gene to assess the editing frequency of the CRISPR/Cas9 system in *L. kaempferi* and explore the influence of endogenous and exogenous U6 promoters on gene editing. Our main goal in this study was to establish a CRISPR/Cas9-mediated gene-editing system for *L. kaempferi* and develop a powerful tool for an *L. kaempferi* gene function study and its genetic improvement.

## 2. Results

### 2.1. Identification of L. kaempferi U6 Genes and Cloning of Their Promoters

**Figure 1:**
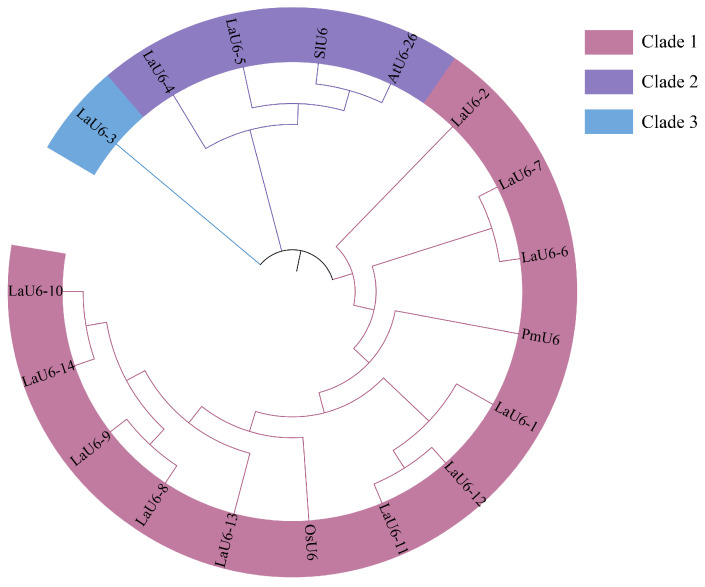
Phylogenetic tree analysis of *U6* genes. Phylogenetic tree analysis was performed using *U6* gene sequences of *Arabidopsis* (*AtU6-26*), *Pseudotsuga menziesii* (Mirb.) Franco (*PmU6*), *Solanum lycopersicum* (*SlU6*), *Oryza sativa* (*OsU6*), and *L. kaempferi* (*LaU6-1*~*LaU6-14*). Different shading colors indicate different clades.

Using the *AtU6-26* and *PmU6* gene sequences as references, 14 *L. kaempferi U6* genes (*LaU6*) were identified from the *L. kaempferi* genome, and they were named *LaU6-1*~*LaU6-14* (Table S1). The 14 *LaU6* gene sequences exhibited high similarity to the *AtU6-26* and *PmU6* gene sequences (Figure 1, Table S1). Based on phylogenetic tree analysis, 11 *U6* genes of *L. kaempferi* belong to the same clade as *PmU6* of *Pseudotsuga menziesii* (Mirb.) Franco and *OsU6* of *Oryza sativa* (clade 1). The *LaU6-4* and *LaU6-5* genes of *L. kaempferi* belong to the same clade as *SlU6* of *Solanum lycopersicum* and *AtU6-26* of *Arabidopsis* (clade 2). *LaU6-3* belongs to clade 3 alone.

**Figure 2:**
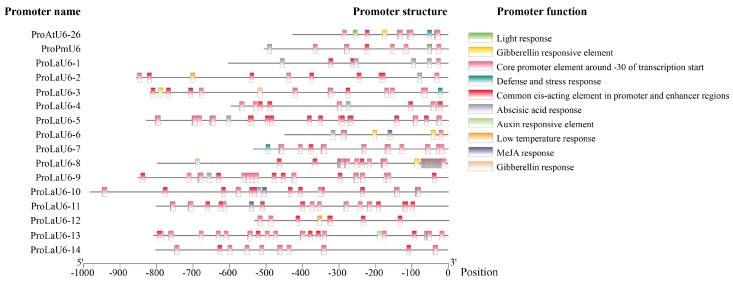
Cis-element analysis of the sixteen U6 promoters. Cis-element analysis was performed using U6 promoter sequences of *Arabidopsis* (ProAtU6-26), *Pseudotsuga menziesii* (Mirb.) Franco (ProPmU6), and 14 *L. kaempferi* (ProLaU6-1~ProLaU6-14). Different shading colors indicate different elements.

Afterward, primers were designed to clone the promoter sequences of these 14 *LaU6* genes, and they were named ProLaU6-1~ProLaU6-14 (Tables S2 and S3). These promoter sequences comprised different cis-elements (Figure 2). In addition, hormone-related cis-elements also existed in most of these promoters (Figure 2).

### 2.2. Vector Construction for the CRISPR/Cas9-Mediated Gene-Editing System in L. kaempferi

**Figure 3:**
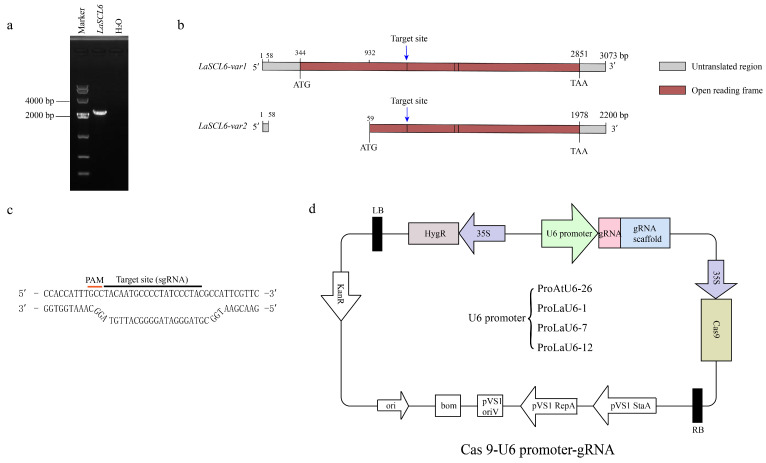
*LaSCL6* target site and vector diagram. (**a**) Electrophoretic map of fragments shared by *LaSCL6-var1* and *LaSCL6-var2*. The common fragment of *LaSCL6-var1* and *LaSCL6-var2* is 2157 bp. (**b**) Target site location and cDNA sequence diagram of *LaSCL6*. (**c**) Target site diagram. The position of the red line indicates the base sequence of the PAM site, and the position of the black line indicates the sequence of the target site. (**d**) Vector diagram. The U6 promoter is replaced by ProAtU6-26, ProLaU6-1, ProLaU6-7, or ProLaU6-12, separately.

PCR was used to amplify the common fragments of *LaSCL6-var1* and *LaSCL6-var2* (Figure 3a), which were then sequenced as the reference. Crispr P 2.0 was employed to design the target sites of *LaSCL6-var1* and *LaSCL6-var2* (Figure 3b,c) [28]. We constructed *LaSCL6* gene-editing vectors; the expression of sgRNA was driven by the ProAtU6-26, ProLaU6-1, ProLaU6-7, and ProLaU6-12 promoters, and the expression of SpCas9 was driven by the CaMV35S promoter (Figure 3d).

### 2.3. Creation of Stable L. kaempferi Transformation Material

**Figure 4:**
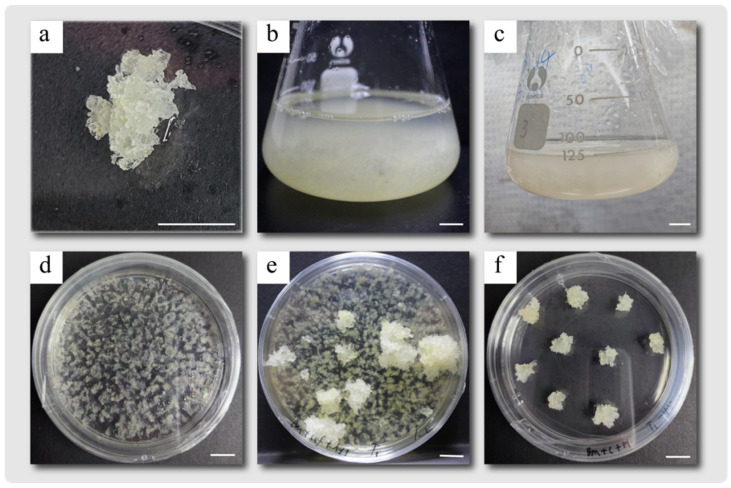
Flow chart of *Agrobacterium*-mediated CRISPR/Cas9 gene editing genetic transformation technique in *L. kaempferi*. (**a**) Embryogenic callus was cultured on solid proliferation medium. (**b**). The embryonic callus was suspended in the liquid proliferation medium. (**c**) Infection. (**d**) Co-culture. (**e**) A round of screening culture. (**f**) Second round of screening culture. Bar = 1 cm.

**Figure 5:**
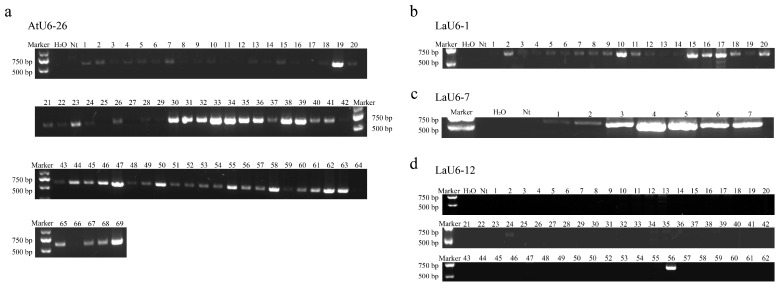
Molecular verification of the Cas9 gene in resistant cell lines. (**a**–**d**) represent agarose gel electrophoresis of Cas9 gene PCR product. The genomic DNA was extracted from resistant callus obtained from genetic transformation of AtU6-26, LaU6-1, LaU6-7, and LaU6-12 recombinant vectors separately. The numbers represent the different cell lines of the resistant callus. Nt indicates non-transgenic material.

Recombinant vectors containing SpCas9 and one sgRNA expression cassette were transformed into an embryonic callus of *L. kaempferi* using the *Agrobacterium*-mediated genetic transformation technique (Figure 4). After infection, co-culture, recovery culture, and screening culture, a different number of resistant cell lines for each vector was obtained (Figure 4). After the genetic transformation of ProAtU6-26, ProLaU6-1, ProLaU6-7 and ProLaU6-12 recombinant vectors, 69 (Figure 5a), 20 (Figure 5b), 7 (Figure 5c), and 62 (Figure 5d) resistant cell lines were obtained (Table 1), respectively.

Then, we used these resistant cell lines (a total of 158 cell lines) for molecular verification and detected the fragments of the Cas9 gene in some resistant cell lines (a total of 90 cell lines) (Figure 5, Table 1). Moreover, the transgene frequencies of the transgenic materials ProAtU6-26, ProLaU6-1, ProLaU6-7, and ProLaU6-12 were 88%, 100%, 100%, and 3%, respectively (Figure 5, Table 1).

### 2.4. CRISPR/Cas9-Mediated LaSCL6 Mutations

**Figure 6:**
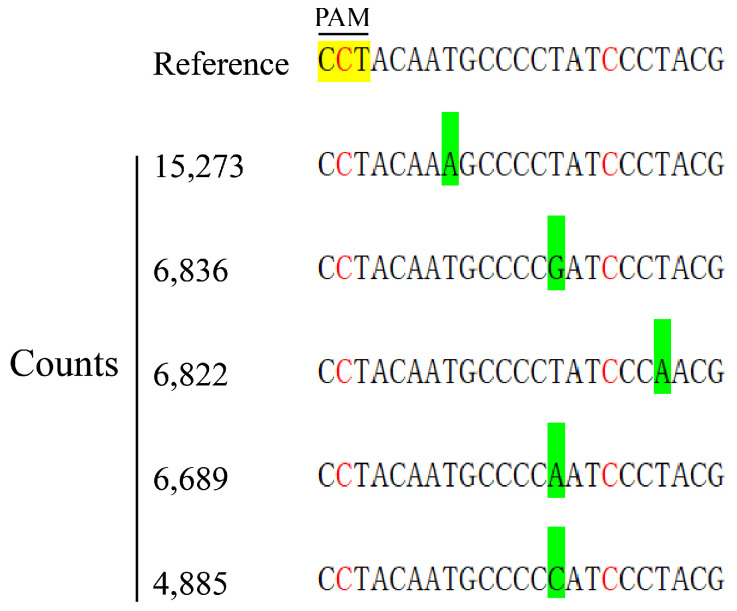
Nucleic acid sequence of *LaSCL6* after gene editing in transient transformation materials. The yellow background indicates the PAM site; the green background indicates a base substitution; the red font indicates SNP site. The numbers in the figure indicate the number of reads in the high-throughput sequencing results.

The high-throughput sequencing analysis showed that the gene-editing frequency was 0.82% (Table S4). The mutation type of the *LaSCL6* gene was base substitution and a total of 5 base substitution types were detected, among which 4885–15,273 reads were detected (Figure 6).

**Figure 7:**
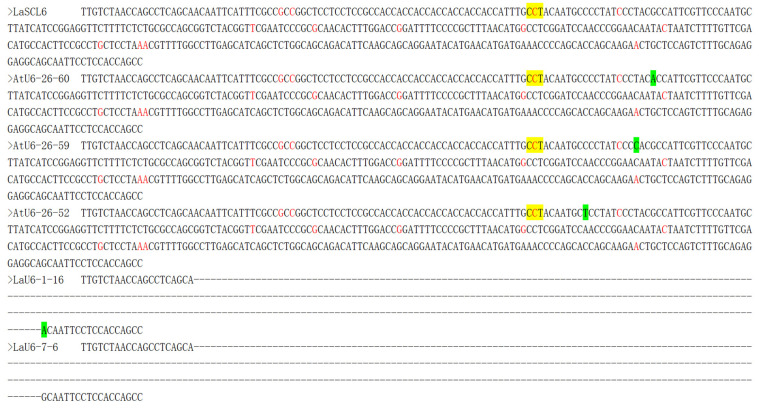
Nucleic acid sequence of *LaSCL6* after gene editing in stable transformation materials. The yellow background in the figure indicates the PAM site; the green background indicates a base substitution; the red font indicates SNP site; and the dashed lines indicate the deleted fragments of nucleic acid sequence.

The Sanger sequencing analysis showed that the gene-editing frequencies of the transgenic materials ProAtU6-26, ProLaU6-1, and ProLaU6-7 were 4.92%, 6.25%, and 14.29%, respectively (Table 1). Three single-base substitutions were detected in three of sixty-one ProAtU6-26 transgenic cell lines, deletion of *LaSCL6* gene fragment (369 bp) was detected in one of sixteen ProLaU6-1 and one of seven ProLaU6-7 transgenic cell lines, respectively, and no mutation was detected in two ProLaU6-12 transgenic cell lines (Table 1, Figure 7). We analyzed the predicted protein sequence of the edited *LaSCL6* and found that the single-base substitution did not alter the LaSCL6 protein sequence, but the base deletion led to the deletion of amino acid sequences (Figure S1).

## 3. Discussion

The CRISPR/Cas9 system is extensively utilized in editing plant genomes. The genome-editing frequency relies on the expression levels of sgRNA and Cas9 [29,30,31,32]. In plant gene-editing systems, sgRNA is typically driven by U6 or U3 promoters, and the editing frequency can be enhanced when sgRNA is driven by the native promoter. When the *GhU6* promoter drives sgRNA expression in *Gossypium hirsutum* L. (cotton), the gene-editing frequency of transgenic materials is increased by 4–6 times [33]. When VvU3/U6 promoters drive sgRNA expression in *Vitis vinifera* L. (grape), the gene-editing frequency of transgenic materials is also significantly increased [34]. In this study, when ProLaU6-1 and ProLaU6-7 drove sgRNA expression, the gene-editing frequency of transgenic materials also increased, further showing the necessity of using the native promoter. Notably, when the ProLaU6 promoters drove sgRNA expression, large fragments of the *LaSCL6* nucleic acid sequence were missing, resulting in changes in the predicted LaSCL6 protein sequences (Figure S1), and when the ProAtU6-26 promoter drove sgRNA expression, single-base substitution was examined in the transgenic materials, with no change in the predicted LaSCL6 protein sequence (Figure S1). These data show that using the native promoter can improve gene-editing efficiency.

In larch, the high-throughput sequencing of the transiently transformed protoplasts shows that the gene-editing frequency is only 1–5% [35]. In this study, high-throughput sequencing of the transiently transformed callus showed that the editing frequency was 0.82% (Table S4). However, the gene-editing frequency in the stably transformed callus can reach 14.29% (Table 1). In transient transformation, T-DNA strands are transiently expressed in the nucleus without being integrated into the genome [36]. Cas nuclease mRNAs and sgRNAs are transcribed in the nucleus, and the mRNAs are then transferred to the cytoplasmic matrix to function [37]. We speculate that the transfer of gene-editing systems between the nucleus and cytoplasm during transient transformation affects the editing frequency [37,38,39].

It is important to note that SNPs may restrict or even cancel CRISPR/Cas9-mediated genome editing [40]. In poplars, SNPs have been identified to change the PAM site from NGG to NGA, rendering the genome completely resistant to editing [41]. Hence, the gene-editing frequency in this study might also be influenced by SNPs because multiple SNPs existed in *LaSCL6* (Table S5), and one SNP existed at the PAM site [42] (Figure 7). A more detailed analysis of the effect of SNP on the editing efficiency of the CRISPR/Cas9 system in *L. kaempferi* would be beneficial. Therefore, it is advisable to avoid SNPs when designing PAM and sgRNA sites.

In this study, the transgene frequency ranged from 3% to 100%, showing that the genetic transformation system was still unstable. The transgene frequency is influenced by several factors, including the concentration of the *Agrobacterium* solution, infection time, acetosyringone concentration, and antibiotic concentration [43,44,45]. Further research is still needed to determine the reason for the transformation system instability.

Somatic embryogenesis offers an innovative approach for the rapid production of a commercially viable hybrid genotype of larch. However, the regeneration efficiency of larch is relatively low. Editing genes related to the regeneration efficiency of larch will be a hot research topic. We plan to use the phytoene desaturase gene to further improve the gene editing technology. In the future, genes in larch will be edited more purposefully to explore gene function and the mechanism of regeneration and improve tree traits.

## 4. Materials and Methods

### 4.1. Cloning of U6 Promoter and SCL6 from L. kaempferi

The published sequences of *A. thaliana AtU6-26* (AT3G13855) and *Pseudotsuga menziesii* (Mirb.) Franco *PmU6* (MW757988.1) (Table S1) were used to search the *L. kaempferi* genome [46], and the *L. kaempferi U6* gene sequences were identified. A fragment of about 800 bp of the *U6* gene upstream was selected as the *U6* gene promoter, and primers were designed for promoter cloning.

The genomic DNA was extracted from the *L. kaempferi* embryonic callus with a plant genome DNA extraction kit (ZOMANBIO; Beijing, China). The *L. kaempferi* U6 promoter sequence was amplified with KOD high-fidelity DNA polymerase and specific primers (Table S2). The PCR products were cloned into the pEASY-T1 vector and then sequenced. The recombinant vectors were extracted and stored at −80 °C for later use.

The phylogenetic tree of the U6 genes (*AtU6-26*, *PmU6*, *OsU6*, *SlU6*, and *LaU6-1~LaU6-14*) was then constructed by MEGA software version 11 using the neighbor-joining (NJ) approach. The phylogenetic tree was visualized and edited using the interactive Tree Of Life (iTOL) tool (https://itol.embl.de/, accessed on 10 November 2024). The cis-elements in promoters were predicted using PlantCARE (https://bioinformatics.psb.ugent.be/webtools/plantcare/html/, accessed on 1 November 2024), while the visual representation of these cis-elements was created using the TBtools software version 2.142.

Total RNA was extracted with an RNA extraction kit (TRANS; Beijing, China) and reverse-transcribed into cDNA with a reverse-transcription kit (TRANS; Beijing, China). The primers 5′-CAAAATCTGGTGGTAATGGAAGATTTG-3′ and 5′-AAGAAGCGAAGAAGCAGACGAGCTT-3′ were used to clone the *LaSCL6* sequence fragment, which is common for two *LaSCL6* alternative splicing forms (*LaSCL6-var1* and *LaSCL6-var2*).

### 4.2. Vector Construction and Genetic Transformation

The CRISPR/Cas9 vector system in this study was modified with the pCAMBIA1305.1 vector as the backbone, including a codon-optimized SpCas9 expression cassette driven by the CaMV35S promoter, hygromycin as the selection marker, and a sgRNA expression cassette. The vector construction was improved based on the method proposed by Liu et al. [28] Genscript Biotech Inc. synthesized the DNA fragment containing an OMIGA translation enhancer followed by rice codon-optimized Cas9 with two OsHAT NLSs on each end. The fragments of sgRNA expression cassettes driven by different U6 promoters were obtained through overlap PCR amplification and then assembled onto the vector backbone using KpnI (5′-GGTAC-3′) and EcoRI (5′-GAATTC-3′). The resulting plasmid was sequenced to be correct and then transformed into the *Agrobacterium tumefaciens* strain GV3101. All the primers used are listed in Table S2.

The methods for transient transformation [43] and stable transformation [1] are referred to in previous studies. The vector containing *Arabidopsis* U6 promoter ProAtU6-26 was used for transient transformation, and the vector containing ProAtU6-26 or one of the three *L. kaempferi* U6 promoters (ProLaU6-1, ProLaU6-7, and ProLaU6-12) was used for stable transformation.

### 4.3. Mutation Detection in Transient Transformation Materials

Transient transformation materials were used to extract genomic DNA with a plant genome DNA extraction kit (ZOMANBIO; Beijing, China). The quality of the DNA was determined via agarose gel electrophoresis, and the DNA concentration was measured with a spectrophotometer. With DNA as the template, the primers 5′-TCCCACATTGTCTAACCAGCC-3′ and 5′-GCGGGATTCGAACCGTAGAC-3′ were used for PCR amplification, and the PCR products were subjected to high-throughput sequencing. The sequencing data were compared with the *LaSCL6* sequences, and the gene-editing frequency in the transient transformation materials was then analyzed.

### 4.4. Mutation Detection in Stable Transformation Materials

Resistant cell lines were used for genomic DNA extraction with a plant genome DNA extraction kit (ZOMANBIO; Beijing, China). With DNA as the template, the primers 5′-GAACCAGACCACCCAGAAGGGCC-3′ and 5′-CGGACACGAGCTTGGACTTGAGGG-3′ were used for PCR amplification of the Cas9 gene fragment (596 bp) to determine the transgene frequency, and the primers 5′-ATCTGCATCTGCGGCTCATTTC-3′ and 5′-GGCTGGTGGAGGAATTGCTGCC-3′ were used for PCR amplification and sequencing to determine the editing frequency. The PCR products were cloned into the *pEASY*^®^-Blunt Zero Cloning Vector and then transformed into the *Trans*1-T1 *Escherichia coli* strain. Subsequently, 50 clones from each transgenic cell line were selected for Sanger sequencing.

## 5. Conclusions

In summary, our successful application of the CRISPR/Cas9 system in *L. kaempferi* facilitates trait improvement through the direct manipulation of the *L. kaempferi* genome and enables the introduction of new traits as well as the enhancement of existing ones.

## Figures and Tables

**Table 1:** Transgene frequency and gene editing frequency.

Construct	Number of Resistant Cell Lines	Number of Transgenic Cell Lines	Transgene Frequency (%)	Number of Transgenic Cell Lines Tested for Genetic Mutation	Number of Transgenic Cell Lines with Mutation	Editing Frequency (%)
AtU6-26	69	61	88	61	3	4.92
LaU6-1	20	20	100	16	1	6.25
LaU6-7	7	7	100	7	1	14.29
LaU6-12	62	2	3	2	0	0
Total	158	90	-	86	5	-

“-” indicates no analysis.

## Data Availability

The data presented in this study are available upon reasonable request from the corresponding author.

## Supplementary Materials

The following supporting information can be downloaded at: www.mdpi.com/article/10.3390/plants14010045/s1, Figure S1: Protein sequence after gene editing in *LaSCL6*; Table S1: *L. kaempferi U6* gene sequence identified in this study; Table S2: Primer sequences for ProU6 promoter cloning and vector construction; Table S3: U6 promoter sequences cloned in this study; Table S4: High-throughput sequencing of *LaSCL6* in larch; Table S5: Statistical table of SNPs in the *LaSCL6* in 158 published larch transcriptomes.
