# Supplementary material for: Identification of U6 Promoter and Establishment of Gene-Editing System in Larix kaempferi (Lamb.) Carr
Source: Plants (Basel). 2024 Dec 26;14(1):45. doi: 10.3390/plants14010045 (PMC11722980; doi:10.3390/plants14010045)
Supplement: Supplementary file 1 [file plants-14-00045-s001.zip › plants-3374621-supplementary/plants-3374621-figures.pdf]

Figure S1

```
>LaSCL6      MNGMLSRTSGNSLPPAQLKHQILQSKTSPVQAQKRHCKSLSRSGGEIEPTSVLDIRSPSPTSTLSSSLGGSSESAGAVAAVSGGLLCEAVSDGGGGGSGSGGAETLLLESSGKEAFNNGGGSTNSPARWYPNRGSSGGSGRE
GFGWRLNNGEQQPGGGGMLEGKSEVKKEEPQQQQQQQSRAEEWGPGCGGGPAGVMEDLESMLLESGAGAPDQSLMRWLLGEIEDPKDLPPPQIKANTSGGSASAAHFEDPSIEPNFGDPVAFSFSSNNISDVIAPPQPAPPPSSFRAPYPTLSNQ
PQQQFISPPAPPPPPPPPPPPPFAYNAPIPTPFVPNAYHPEVLFSAPAVYGSNPAQHFGPDFPRFNMASDPTRNNNNLLFDMPLPPAPKRFGLEHQLWQQTFKQQEYMNMMKPQHQQELLQSLQRRQQFLHQPPPPPPHHQQLRQKAIVNNTLKVES
SGAAAEEVQVIVEQLLKAAEAVELGNLDHAQAILARLNQHLSPLGKPLHRAAFYFKEALASRILNATASTTGGDNRNATGSGTGTSNISPLDMVHKISAYKSFSEASPLAQFAHFTANQALLEALDGAEAIHIIDFEIGLGGQWASFLQELAVKL
GGAPPVRLTALGTSASSSLELHLTRDNLCNFAKQLNVPFEFELLHLDRIESLTLREREAVAVNLSLLPSTFTSLDSISRLLNLIKNLSPRAVVAVDAETTASAAATSPAASFVHHFLEALQFYSFMFDSLDAVNINMDAVHKIEKFLLAPKIDAT
ISSAAKPPWKTLFASAGFSPVAFSNFTETQAEYLIQRLHGRGFEVLKAHTALLLGWQGRPLVSATAWRCGPPP*
>AtU6-26-52  MNGMLSRTSGNSLPPAQLKHQILQSKTSPVQAQKRHCKSLSRSGGEIEPTSVLDIRSPSPTSTLSSSLGGSSESAGAVAAVSGGLLCEAVSDGGGGGSGSGGAETLLLESSGKEAFNNGGGSTNSPARWYPNRGSSGGSGRE
GFGWRLNNGEQQPGGGGMLEGKSEVKKEEPQQQQQQQSRAEEWGPGCGGGPAGVMEDLESMLLESGAGAPDQSLMRWLLGEIEDPKDLPPPQIKANTSGGSASAAHFEDPSIEPNFGDPVAFSFSSNNISDVIAPPQPAPPPSSFRAPYPTLSNQ
PQQQFISPPAPPPPPPPPPPPPFAYNAPIPTPFVPNAYHPEVLFSAPAVYGSNPAQHFGPDFPRFNMASDPTRNNNNLLFDMPLPPAPKRFGLEHQLWQQTFKQQEYMNMMKPQHQQELLQSLQRRQQFLHQPPPPPPHHQQLRQKAIVNNTLKVES
SGAAAEEVQVIVEQLLKAAEAVELGNLDHAQAILARLNQHLSPLGKPLHRAAFYFKEALASRILNATASTTGGDNRNATGSGTGTSNISPLDMVHKISAYKSFSEASPLAQFAHFTANQALLEALDGAEAIHIIDFEIGLGGQWASFLQELAVKL
GGAPPVRLTALGTSASSSLELHLTRDNLCNFAKQLNVPFEFELLHLDRIESLTLREREAVAVNLSLLPSTFTSLDSISRLLNLIKNLSPRAVVAVDAETTASAAATSPAASFVHHFLEALQFYSFMFDSLDAVNINMDAVHKIEKFLLAPKIDAT
ISSAAKPPWKTLFASAGFSPVAFSNFTETQAEYLIQRLHGRGFEVLKAHTALLLGWQGRPLVSATAWRCGPPP*
>AtU6-26-59  MNGMLSRTSGNSLPPAQLKHQILQSKTSPVQAQKRHCKSLSRSGGEIEPTSVLDIRSPSPTSTLSSSLGGSSESAGAVAAVSGGLLCEAVSDGGGGGSGSGGAETLLLESSGKEAFNNGGGSTNSPARWYPNRGSSGGSGRE
GFGWRLNNGEQQPGGGGMLEGKSEVKKEEPQQQQQQQSRAEEWGPGCGGGPAGVMEDLESMLLESGAGAPDQSLMRWLLGEIEDPKDLPPPQIKANTSGGSASAAHFEDPSIEPNFGDPVAFSFSSNNISDVIAPPQPAPPPSSFRAPYPTLSNQ
PQQQFISPPAPPPPPPPPPPPPFAYNAPIPTPFVPNAYHPEVLFSAPAVYGSNPAQHFGPDFPRFNMASDPTRNNNNLLFDMPLPPAPKRFGLEHQLWQQTFKQQEYMNMMKPQHQQELLQSLQRRQQFLHQPPPPPPHHQQLRQKAIVNNTLKVES
SGAAAEEVQVIVEQLLKAAEAVELGNLDHAQAILARLNQHLSPLGKPLHRAAFYFKEALASRILNATASTTGGDNRNATGSGTGTSNISPLDMVHKISAYKSFSEASPLAQFAHFTANQALLEALDGAEAIHIIDFEIGLGGQWASFLQELAVKL
GGAPPVRLTALGTSASSSLELHLTRDNLCNFAKQLNVPFEFELLHLDRIESLTLREREAVAVNLSLLPSTFTSLDSISRLLNLIKNLSPRAVVAVDAETTASAAATSPAASFVHHFLEALQFYSFMFDSLDAVNINMDAVHKIEKFLLAPKIDAT
ISSAAKPPWKTLFASAGFSPVAFSNFTETQAEYLIQRLHGRGFEVLKAHTALLLGWQGRPLVSATAWRCGPPP*
>AtU6-26-60  MNGMLSRTSGNSLPPAQLKHQILQSKTSPVQAQKRHCKSLSRSGGEIEPTSVLDIRSPSPTSTLSSSLGGSSESAGAVAAVSGGLLCEAVSDGGGGGSGSGGAETLLLESSGKEAFNNGGGSTNSPARWYPNRGSSGGSGRE
GFGWRLNNGEQQPGGGGMLEGKSEVKKEEPQQQQQQQSRAEEWGPGCGGGPAGVMEDLESMLLESGAGAPDQSLMRWLLGEIEDPKDLPPPQIKANTSGGSASAAHFEDPSIEPNFGDPVAFSFSSNNISDVIAPPQPAPPPSSFRAPYPTLSNQ
PQQQFISPPAPPPPPPPPPPPPFAYNAPIPTPFVPNAYHPEVLFSAPAVYGSNPAQHFGPDFPRFNMASDPTRNNNNLLFDMPLPPAPKRFGLEHQLWQQTFKQQEYMNMMKPQHQQELLQSLQRRQQFLHQPPPPPPHHQQLRQKAIVNNTLKVES
SGAAAEEVQVIVEQLLKAAEAVELGNLDHAQAILARLNQHLSPLGKPLHRAAFYFKEALASRILNATASTTGGDNRNATGSGTGTSNISPLDMVHKISAYKSFSEASPLAQFAHFTANQALLEALDGAEAIHIIDFEIGLGGQWASFLQELAVKL
GGAPPVRLTALGTSASSSLELHLTRDNLCNFAKQLNVPFEFELLHLDRIESLTLREREAVAVNLSLLPSTFTSLDSISRLLNLIKNLSPRAVVAVDAETTASAAATSPAASFVHHFLEALQFYSFMFDSLDAVNINMDAVHKIEKFLLAPKIDAT
ISSAAKPPWKTLFASAGFSPVAFSNFTETQAEYLIQRLHGRGFEVLKAHTALLLGWQGRPLVSATAWRCGPPP*
>LaU6-1-16   MNGMLSRTSGNSLPPAQLKHQILQSKTSPVQAQKRHCKSLSRSGGEIEPTSVLDIRSPSPTSTLSSSLGGSSESAGAVAAVSGGLLCEAVSDGGGGGSGSGGAETLLLESSGKEAFNNGGGSTNSPARWYPNRGSSGGSGRE
GFGWRLNNGEQQPGGGGMLEGKSEVKKEEPQQQQQQQSRAEEWGPGCGGGPAGVMEDLESMLLESGAGAPDQSLMRWLLGEIEDPKDLPPPQIKANTSGGSASAAHFEDPSIEPNFGDPVAFSFSSNNISDVIAPPQPAPPPSSFRAPYPTLSNQ
PQQQFL----------------------------------------------------------------------------------------------------------------------------HQPPPPPPHHQQLRQKAIVNNTLKVES
SGAAAEEVQVIVEQLLKAAEAVELGNLDHAQAILARLNQHLSPLGKPLHRAAFYFKEALASRILNATASTTGGDNRNATGSGTGTSNISPLDMVHKISAYKSFSEASPLAQFAHFTANQALLEALDGAEAIHIIDFEIGLGGQWASFLQELAVKL
GGAPPVRLTALGTSASSSLELHLTRDNLCNFAKQLNVPFEFELLHLDRIESLTLREREAVAVNLSLLPSTFTSLDSISRLLNLIKNLSPRAVVAVDAETTASAAATSPAASFVHHFLEALQFYSFMFDSLDAVNINMDAVHKIEKFLLAPKIDAT
ISSAAKPPWKTLFASAGFSPVAFSNFTETQAEYLIQRLHGRGFEVLKAHTALLLGWQGRPLVSATAWRCGPPP*
>LaU6-7-6    MNGMLSRTSGNSLPPAQLKHQILQSKTSPVQAQKRHCKSLSRSGGEIEPTSVLDIRSPSPTSTLSSSLGGSSESAGAVAAVSGGLLCEAVSDGGGGGSGSGGAETLLLESSGKEAFNNGGGSTNSPARWYPNRGSSGGSGRE
GFGWRLNNGEQQPGGGGMLEGKSEVKKEEPQQQQQQQSRAEEWGPGCGGGPAGVMEDLESMLLESGAGAPDQSLMRWLLGEIEDPKDLPPPQIKANTSGGSASAAHFEDPSIEPNFGDPVAFSFSSNNISDVIAPPQPAPPPSSFRAPYPTLSNQ
PQQQFL----------------------------------------------------------------------------------------------------------------------------HQPPPPPPHHQQLRQKAIVNNTLKVES
SGAAAEEVQVIVEQLLKAAEAVELGNLDHAQAILARLNQHLSPLGKPLHRAAFYFKEALASRILNATASTTGGDNRNATGSGTGTSNISPLDMVHKISAYKSFSEASPLAQFAHFTANQALLEALDGAEAIHIIDFEIGLGGQWASFLQELAVKL
GGAPPVRLTALGTSASSSLELHLTRDNLCNFAKQLNVPFEFELLHLDRIESLTLREREAVAVNLSLLPSTFTSLDSISRLLNLIKNLSPRAVVAVDAETTASAAATSPAASFVHHFLEALQFYSFMFDSLDAVNINMDAVHKIEKFLLAPKIDAT
ISSAAKPPWKTLFASAGFSPVAFSNFTETQAEYLIQRLHGRGFEVLKAHTALLLGWQGRPLVSATAWRCGPPP*
```

Figure S1. Protein sequence after gene editing in *LaSCL6*. The red background in the figure indicates an amino acid substitution, and the red dashed lines indicate deletion segments of amino acid.
